# Vertical Migrations of a Deep-Sea Fish and Its Prey

**DOI:** 10.1371/journal.pone.0097884

**Published:** 2014-05-23

**Authors:** Pedro Afonso, Niall McGinty, Gonçalo Graça, Jorge Fontes, Mónica Inácio, Atle Totland, Gui Menezes

**Affiliations:** 1 IMAR - Institute of Marine Research at the University of the Azores, Dept. of Oceanography and Fisheries, Horta, Portugal; 2 LARSyS – Laboratory of Robotics and Systems in Engineering and Science, Lisboa, Portugal; 3 MARICE, Faculty of Life and Environmental Sciences, University of Iceland, Reykjavik, Iceland; 4 IMR - Institute of Marine Research, Bergen, Norway; Scottish Association for Marine Science, United Kingdom

## Abstract

It has been speculated that some deep-sea fishes can display large vertical migrations and likely doing so to explore the full suite of benthopelagic food resources, especially the pelagic organisms of the deep scattering layer (DSL). This would help explain the success of fishes residing at seamounts and the increased biodiversity found in these features of the open ocean. We combined active plus passive acoustic telemetry of blackspot seabream with *in situ* environmental and biological (backscattering) data collection at a seamount to verify if its behaviour is dominated by vertical movements as a response to temporal changes in environmental conditions and pelagic prey availability. We found that seabream extensively migrate up and down the water column, that these patterns are cyclic both in short-term (tidal, diel) as well as long-term (seasonal) scales, and that they partially match the availability of potential DSL prey components. Furthermore, the emerging pattern points to a more complex spatial behaviour than previously anticipated, suggesting a seasonal switch in the diel behaviour mode (benthic vs. pelagic) of seabream, which may reflect an adaptation to differences in prey availability. This study is the first to document the fine scale three-dimensional behaviour of a deep-sea fish residing at seamounts.

## Introduction

The behavioural ecology behind the migrations and habitat use of marine fishes intrigued scientists from the very first forays of ocean discovery, but our knowledge acquired since then has been very much skewed towards inshore fishes and their habitats. In contrast, we know almost nothing on the behaviour of fishes living in the vast depths of the ocean.

It is speculated that some deep-sea fishes are thought to be capable of moving between the flanks and adjacent midwater zones of seamounts - biodiversity rich, underwater mountains in the open ocean - and display vertical migrations of several hundreds of meters [1]–[4]. Such behaviour would allow them to better explore the full suite of benthopelagic food resources that can be found around seamounts. In particular, the huge biomass of pelagic organisms that form the deep (sound) scattering layer (DSL) and which are known to perform large diel vertical migrations [5]. The ability to vertically migrating would be an adaptive behaviour of deep-sea fishes to take advantage of vertically migratory prey. To date, this hypothesis has not been formally tested. Neither has the ability of such fishes to migrate between the various ecological niches at a seamounts' dynamic habitat (benthic versus pelagic, summit versus slope).

More broadly, typifying of the spatial and behavioural ecology of deep-sea fishes has been based on indirect evidence from fishing or echosounding profiles with very few cases of direct, detailed behavioural data [6]. In this paper we present the results of a combined passive and active acoustic telemetry experiment designed to verify that habitat use of highly mobile fishes at seamounts is based on their ability to undergo vertical migrations in the water column and that they do so as a response to environmental conditions and prey availability. We use the blackspot seabream (*Pagellus bogaraveo*) as a model species. This is a schooling, carnivorous sparid whose adults are a major constituent of the meso-benthopelagic fish assemblage over slopes and seamounts of the northeast Atlantic [7], [8]. They rely on the DSL components as a food source [5], [9]. Our study is unique in that it records the three-dimensional individual behaviour of deep-sea fishes and concurrent synoptic data, including the estimated DSL biomass. We hypothesized that sea bream undergoes vertical movements at seamounts, and that they would be correlated with tidal, diel and seasonal rhythms associated with changes in local physical oceanography and prey availability. The latter being of particular interest in view of what is known about the diel vertical migration of the DSL.

## Materials and Methods

### Ethics statement

This study was performed according to national Portuguese laws for the use of vertebrates in research, and the work and tagging protocols approved by the Azorean Directorate of Sea Affairs of the Azores Autonomous region (DRAM/SRRN ref. 24/2010), which oversees and issues permits for scientific activities in the Condor Seamount Marine Protected Area. All procedures followed the guidelines for the use of fishes in research of the American Fisheries Society. The field studies did not involve endangered or protected species, no animals were sacrificed, and procedures for reduction, replacement and refinement were thoroughly adopted.

### Study site

**Figure 1:**
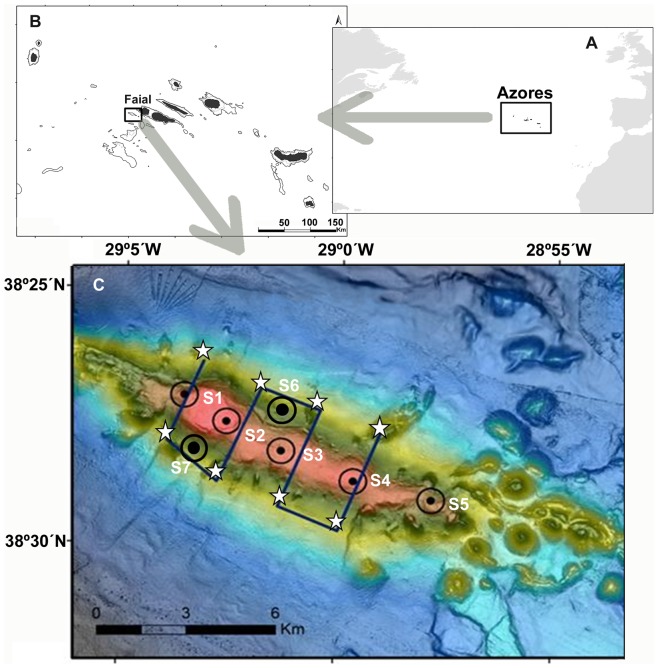
The Condor seamount. Location of the Azores archipelago in the mid-North Atlantic (A) showing the islands (in black) and the 500 m isobath (dark line) (B), and the location of the Condor seamount (C). Also showed are the numbered passive acoustic monitoring stations (black dots) and respective estimated listening range (black rings), the acoustic survey transects (dark line) and the CTD stations (white stars). The four transverse profiles across the seamount used in backscatter analysis (Figure 4, Figures S3 and S4) can be identified from left (T1) to right (T4).

The study was conducted at the Condor seamount in the mid-north Atlantic archipelago of the Azores (Figure 1), an 8 km long elongated volcanic structure that rises steeply (15 to 23°) from depths over 1000 m to around 200 m at its flat summit. Its moderate size and the high diversity, representativeness and good conservation status of the local benthic habitats and biotopes [10], [11], together with its proximity to port (20 nm) and current status as a marine reserve, lends itself as a unique location to study seamount ecology and conservation.

### Active tracking

We actively tracked four adult blackspot seabream at Condor using manual acoustic telemetry to test hypotheses relating to short term patterns of vertical and horizontal behaviour (Table 1). Active tracking consisted of surgically implanting fish with an acoustic transmitter in the peritoneal cavity that emitted an ultrasonic ping every one to two seconds and following them from two small vessels using manual acoustic receivers and logging the GPS positions every 30 minutes when possible [12]. Fish were caught by handlining at the west side of the Condor seamount on the night of June 19th 2012 and tagged with pressure (depth) sensor transmitters rated to 500 m depth (β-V13P-1H VEMCO transmitters, Halifax CAN). In order to reduce the probability and extent of barotraumas and decompression disease, we adopted some simple preventive mitigation techniques. Fish were targeted only from the seamount summit above 150 m and we avoided individuals occupying the seamounts deeper flanks, thereby reducing the absolute rate of change in pressure. The fish were hauled slowly (ca. 0.2 m/second) to promote the natural release of excess gas through the gut and reduce the probability of swim bladder rupture. Time at the surface was minimized (the whole handling procedure typically lasted under 4 minutes) and the fish were quickly released at the site of capture after visual confirmation of their normal behaviour (regular ventilation, horizontal positioning and normal swimming). All fish were tracked for 48 hours during the full moon starting five days after release and for an additional 24 hours during new moon which started 20 days after release. This protocol avoided potentially biased behavioural data typical of post release recovery periods [12] while covering day and night periods in the two extreme moon phases. Fish number 1 (A1) was tracked for an additional 24 hours in the second period since this individual had substantially less detections during the first period.

**Table 1:** Summary information for blackspot seabream tagged with deep-pressure sensor acoustic transmitters and either actively tracked (A) or passively monitored (P) at Condor.

fish ID	length (cm)	days monitored	hours tracked	days detected	speed (km/hour)	distance (km/day)	depth (m)	height (m)
A1	48.5	17	96		0.5	14.8	278(244–524)	45(0–199)
A2	35	17	72		0.5	14.5	270(190–322)	21(0–126)
A3	38.5	17	72		0.3	7.1	178(136–205)	51(0–140)
A4	36	17	72		0.3	7.4	181(151–236)	45(0–148)
P5	49.5	667		526			268(206–337)	35(0–130)
P6	47	667		4			235(149–550)	25(0–90)
P7	38	667		218			223(200–279)	33(0–172)
P8	48.5	631		195			238(205–337)	28(0–114)

Movement and detection statistics report average (minimum – maximum) values. Height refers to the vertical distance from the fish depth to the bottom.

### Passive telemetry

We monitored the long-term, three-dimensional habitat use of four adult blackspot seabream (Table 1) at Condor using passive acoustic telemetry to determine seasonal changes in their horizontal and vertical behaviour. Passive telemetry consisted of tagging each fish with pressure-sensor acoustic transmitters (β-V13P-1L) that emitted a coded ultrasonic ping on average every 120 seconds and continuously monitoring their presence within a 450 m detection radius of omnidirectional acoustic receivers fixed 5 to 30 meters above the seafloor at selected sites along the seamounts crest and flanks [6]. Fish were captured, tagged and released as above on the nights of the 12^th^April and 19^th^ May 2011. The expected battery life of the transmitters was 745 days. We monitored the fish for 22 months from April 13th 2011 to February 2nd 2013 using five receivers (stations 1–5) moored along the seamount summit at 190–250 m bottom depth (Figure 1). Two additional receivers (stations 6 and 7) were kept for a shorter period (April to September 2011) on the flanks of the seamount at 350 and 500 m bottom depth, respectively, to assess if any significant changes in behaviour or detectability occur between the top and the flanks of the bank. Stations were rigged with an acoustic release (AR50/60 SubSeaSonics, San Diego USA) and retrieved every three to six months to download stored information. None of the eight fish (active plus passive telemetry) showed signs of barotrauma or abnormal behaviour [13] when tagged and the fish swam vigorously towards the bottom upon release.

### Environmental variables

A day and a night-time transect were performed concurrently across the seamount during each active tracking period using the RV ‘Archipelago’. Each transect comprised a continuous echo sounder (SIMRAD ES60, 18 KHz) profile followed by the deployment of a CTD sensor array to measure temperature, salinity and dissolved oxygen (SBE 911) from 12 stations along the transect grid (Figure 1). The Nautical Area Scattering Coefficient (NASC/s_A_) was adopted as the acoustic parameter to estimate the backscatter strength and as a proxy for biomass within the water column [14] and was integrated into 10×100 m cells across each transect. Crepuscular periods were defined as one hour before sunrise and after sunset times for the Azores, extracted from the NOAA database.

### Statistical analysis

#### Active tracking

We examined changes in individual vertical distribution across moon phases and day/night periods using a three way ANOVA with a random effect (Fish ID) to account for changes in depth (i.e. vertical migration) or distance to the bottom (i.e. benthic versus benthopelagic behaviour). Each fish detection was associated with the subjacent or closest DSL profile, including depth and backscatter intensity, to visually determine fish position in respect to the DSL. We also estimated the three-dimensional (3D) 95% and 50% Kernel Utilization Distributions (KUDs) calculated from raw xyz positions as representative estimates of the short-term seabream core activity and home range areas, respectively. Three-dimensional KUDs combining all detections were calculated for each individual using the bespoke scripts [15] and used to calculate the percent overlap between individual areas.

#### Passive telemetry

Data from the four individuals were examined to assess possible diel and seasonal changes in vertical behaviour across the 22 month monitoring period. The time series was pre-screened to remove spurious detections [12] and a one-way ANOVA was used to test for differences in depth between detections at deeper flanking receivers (St6 and St7) versus core summit receivers (St1–St5) while both sets of receivers were active (six months). The depth variation in the long term (daily) and short term (hourly) time series at core receivers was then examined using Fourier Fast Transform (FFT) analysis to investigate synchrony with the moon phase or diel migratory behaviour. A generalized additive model (GAM) was used to assess changes in the 1) depth and 2) behavioural state (benthic/pelagic) on both a long (monthly) and short (daily) term basis across the core stations (details in Electronic Supplement). The behavioural state was determined by comparing the fish depth with the average bottom depth (plus two standard deviations) within the 450 m detection range of each station calculated with high-resolution (5 m) bathymetry [11]. Fish detections within this depth range were considered to be characteristic of a benthic behavioural state, detections made outside this depth range were considered characteristic of a pelagic state. The GAM models for 1) depth and 2) behavioural state (distance above seafloor) of passively monitored fish are of the form: 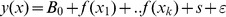
(1)

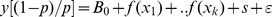
(2)where *y*(*x*) represents the Gaussian link function for the depth detections of the fish and y[(1−*p*)/*p*] represents the binomial link function for behavioural state of the fish, *f*(*x_k_*) represents the smoothing functions of the covariates (the identity of these vary between models), *s* represents the potential inclusion of a parametric term (in this instance station) and *B_0_* and *ε* are the intercept and error term, respectively.

## Results

### Active telemetry

**Figure 2:**
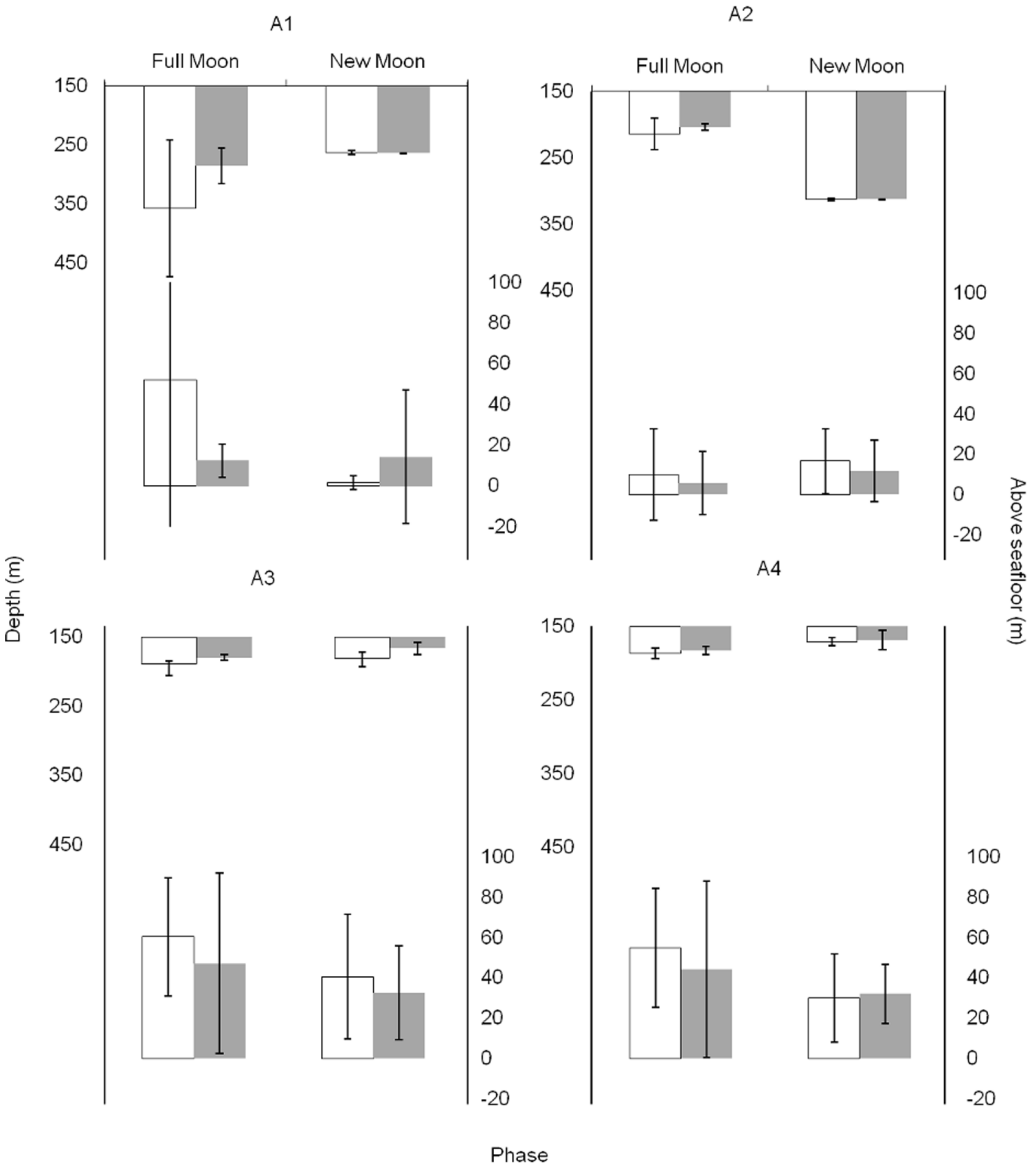
Active telemetry: vertical behaviour from versus moon and diel phase. The average (± 95% confidence interval) depth (upper portion of graphs) and distance above seafloor (lower portion of graphs) of the four individuals actively tracked across the Condor seamount is shown between each lunar period (full moon vs. new moon) and time of day (day/white vs. night/grey).

Each of the four fish generated a minimum of 36 hours tracking that spanned 17 days (Table 1), during which they displaced an average 11 km/day at an average 0.41 km/hour but with substantial individual differences in horizontal movements. Fishes A3 and A4 remained in close proximity to each other and in the same area during both sampling periods, whereas A2 and A1 used more extensive areas (up to 7 km long) in at least one period and different areas among periods (Figure S1). Vertical behaviour matched this pattern, with A3 and A4 utilizing shallower waters at the summit compared to A2 and, especially, A1 which ventured deeper into the slopes (Figure 2). These two-dimensional patterns are well-differentiated in the three-dimensional home range (Figure S2), with a remarkable 60% overlap between the 95% 3D-KUD volumes of individuals A3 and A4 (Table S1). Fish were found deeper during daytime than at night, at least during the first period, with three of these individuals also found deeper during full moon than new moon (Figure 2). However, these depth changes were only found to be significant between moon phases (*F*_1,296_ = 33.2; *p*<0.001) and there was no significant interaction between daytime period and moon phase. Conversely, there was no clear change in height of the fish above the seafloor between either daytime or moon phases (Figure 2).

**Figure 3:**
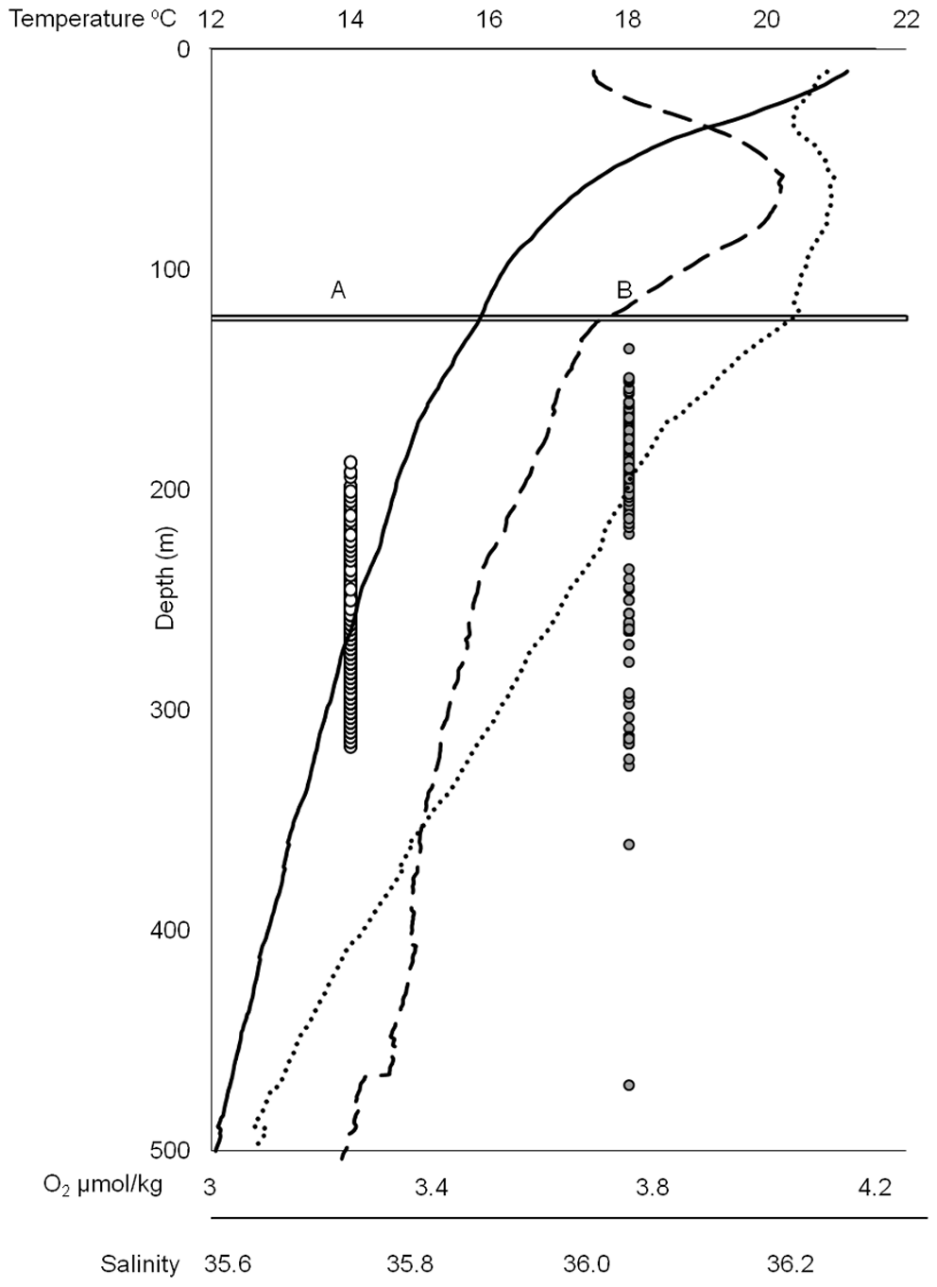
Active telemetry: vertical behaviour from versus environmental conditions. Vertical profiles show composite CTD casts across both phases (temperature - black line; O2 -dashed line; salinity - dotted line), the horizontal line shows the average thermocline/pycnocline position at 120 m. The circles display the vertical positions of the individual detections made during July 2012 by the passive detectors (white circles) and the detections made during both phases for the actively tracked fish (grey circles).

The CTD casts showed strong homogeneity in the water column structure across the seamount which persisted between cruises, with correlations >0.9 for temperature, salinity and O^2^ across all profiles at depths bellow 100 m. The comparison of fish depths versus an average composite of the three profiles indicated that all fish used the water column above the seamount down to 534 m, but never at or shallower than the lower thermocline/pycnocline at ∼120 m, when dissolved oxygen stabilizes (Figure 3).

**Figure 4:**
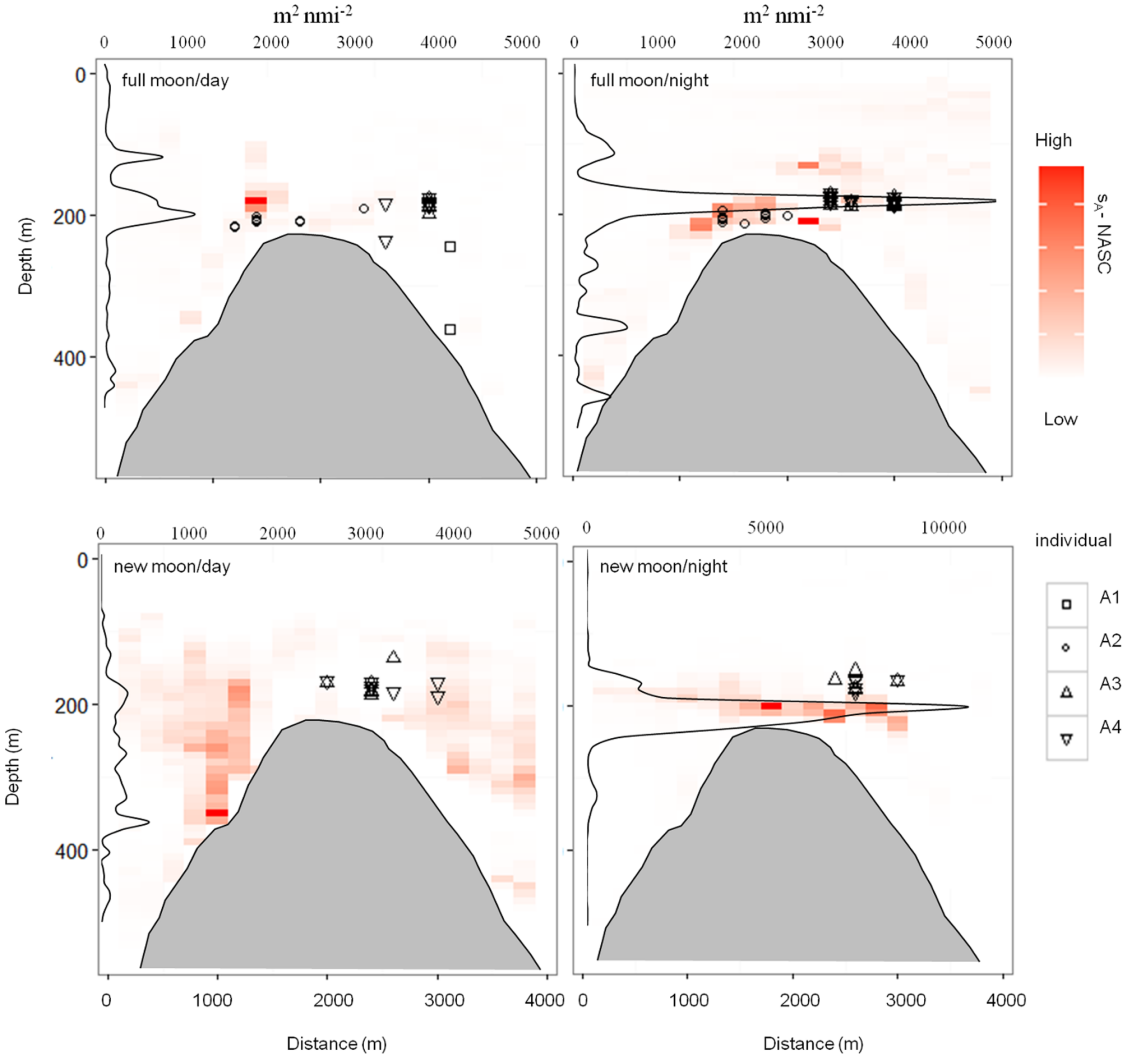
Active telemetry: vertical behaviour versus potential prey. The depth locations of the four individuals during the first, full moon active tracking period overlaid on the closest echosounder transect collected during the same day/night time period. Backscatter strength is binned into 10×100 m cells with the higher backscatter strength represented by increasingly darker red cell. The vertical profile of the backscatter strength is also shown with the maximum backscatter strength for each 10 m vertical bin is also shown. Note: For brevity, only transect one (west summit) is shown, where the majority of detections took place.

The vertical positioning and strength of the DSL over the seamount varied strongly across the seamount during periods, with the greatest day vs. night difference in backscatter sA coefficient found in the western transects (1 and 2), where most detections took place (Figure 4, Figures S3 and S4). The DSL was often vertically heterogeneous during daytime but maximum S_A_ values were always seen on the flanks below 250 m. In contrast, night-time DSL was concentrated within a thin horizontal band about 20 m above the seamount summit. Night-time maximum sA values were between four to ten times greater than daytime values. Although changes in depth for each individual fish did not vary significantly between time of day (Figure 2), this night-time strengthening of the DSL at the seamount crest was broadly spatially coincident with the bulk of night-time fish detections in both the full moon and new moon tracking periods (Figure 4, Figures S5 and S6). This matching pattern was typical of daytime observations, when fish positions and DSL patches were often displaced.

### Passive telemetry

**Figure 5:**
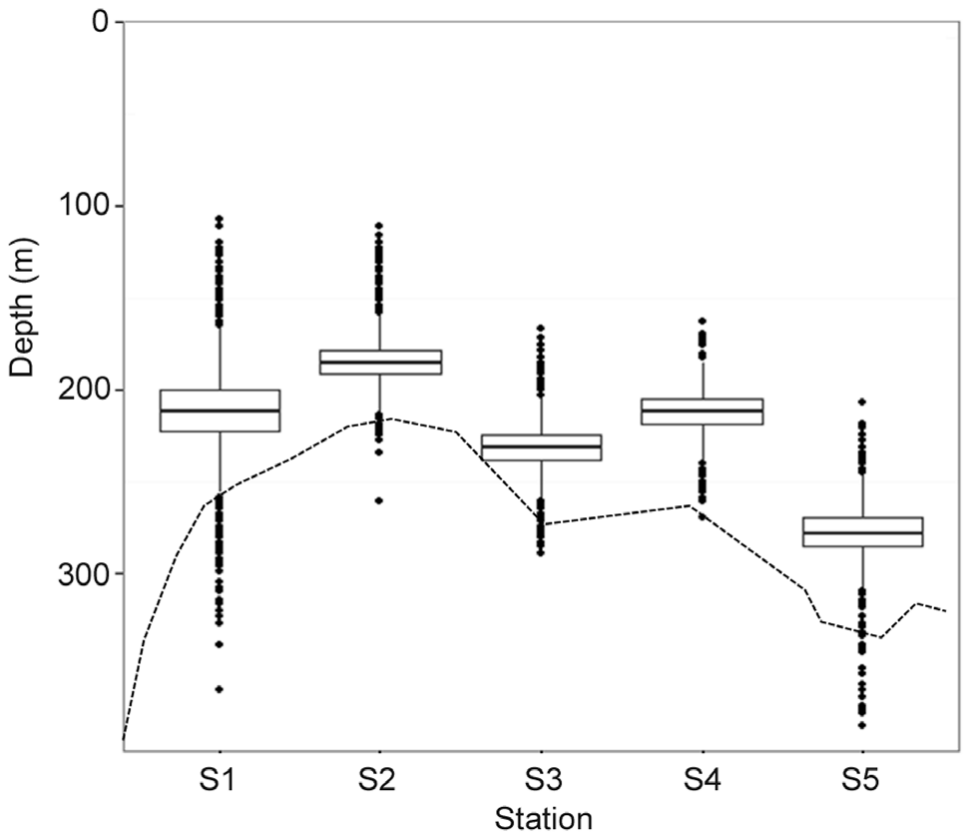
Passive telemetry: fish vertical habitat envelope vs. the seamount bottom profile. Boxplot of the depths (average ± SD and outliers) recorded at each of the five main stations overlaid on the corresponding transverse section of the Condor seamount (dashed line).

Fish P8 was detected until 195 days after tagging, whereas P5 and P7 were still being heard when the experiment was terminated nearly two years later. P6 detection stopped at ∼day 8 and was omitted from the analysis. Similarly to actively tracked individuals, 95% of all passive detections were between 200 and 300 m depth and the distance above seafloor rarely exceeded 50 m (Table 1, Figure 3). Across the seamount, fish seemed to accompany the general bottom profile but maintained the same general vertical habitat envelope in relation to the bottom (Figure 5).

**Figure 6:**
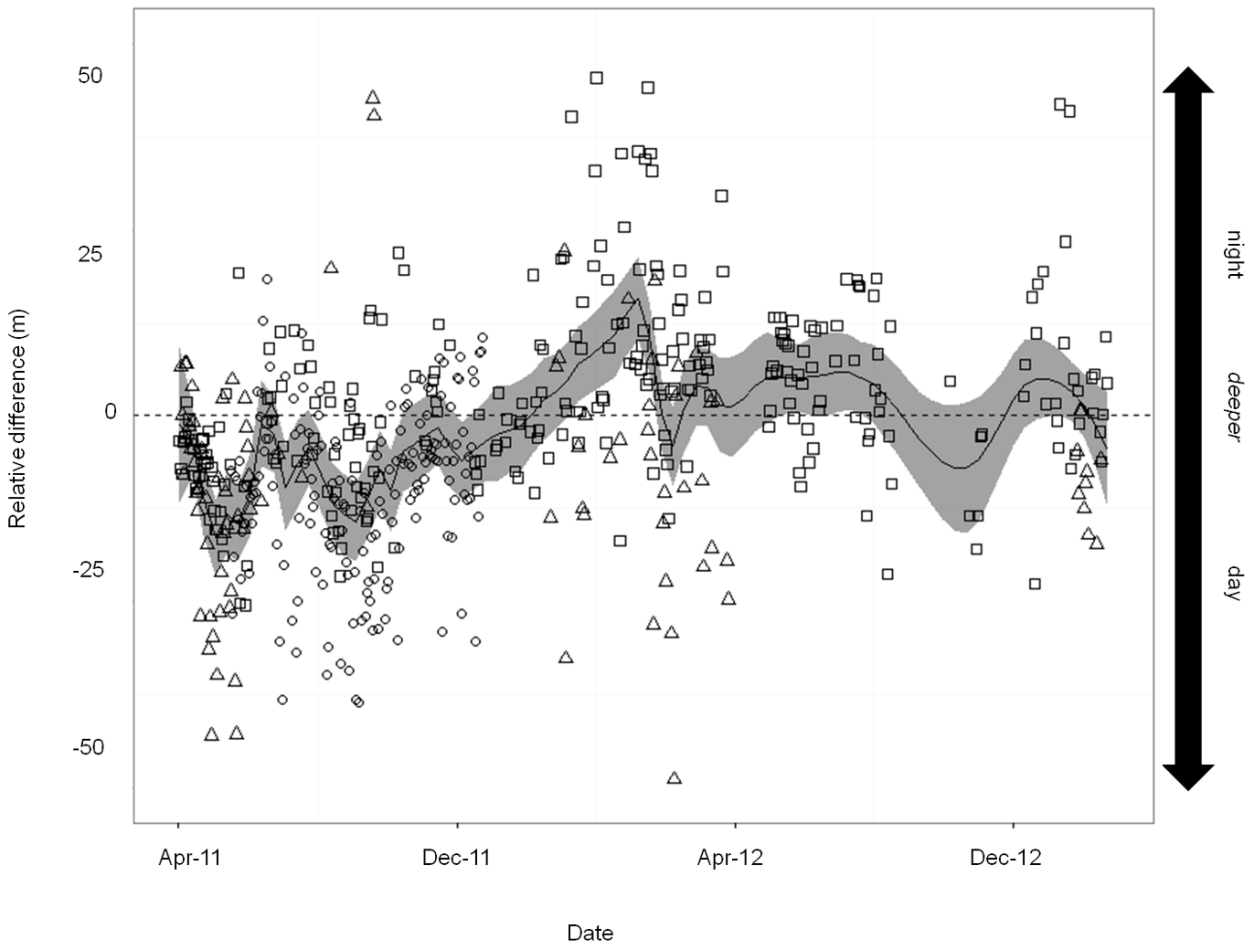
Passive telemetry: seasonal shifts in diel average depth. The relative difference in the average daily period (night and day) depth for each of the three individuals (assigned with different markers - see Fig 6) across the monitoring period. Values below the x origin indicate days where daytime depths were deepest while values above the x origin indicate days where night time depths were the deepest.

We found an annual cycle from shallower depths in the winter/spring months at ∼200 m to increasing depths through summer up to ∼350–500 m in autumn (Figure S5). Fish depth was significantly shallower among the five core stations than the two deeper flanking stations (*F*_1,51045_ = 1.1×10^−4^; *p*<0.001), where average depths also increased through the summer months (Figure S6). The shorter-term periodicity of individual vertical behaviour as shown by FFT analysis was dominated by diel patterns, particularly at the ∼6 hr and ∼24 hr periods. There was no evidence of any regular long-term lunar cycle (Figure S7). These depth fluctuations were within a 25–50 m vertical band, although fish were found much deeper than the seamount summit at distinct periods. Interestingly, fish tended to be deeper during the day than during the night in 2011 (May onwards) but switched to shallower daytime depths through the first half of 2012 (Figure 6).

**Figure 7:**
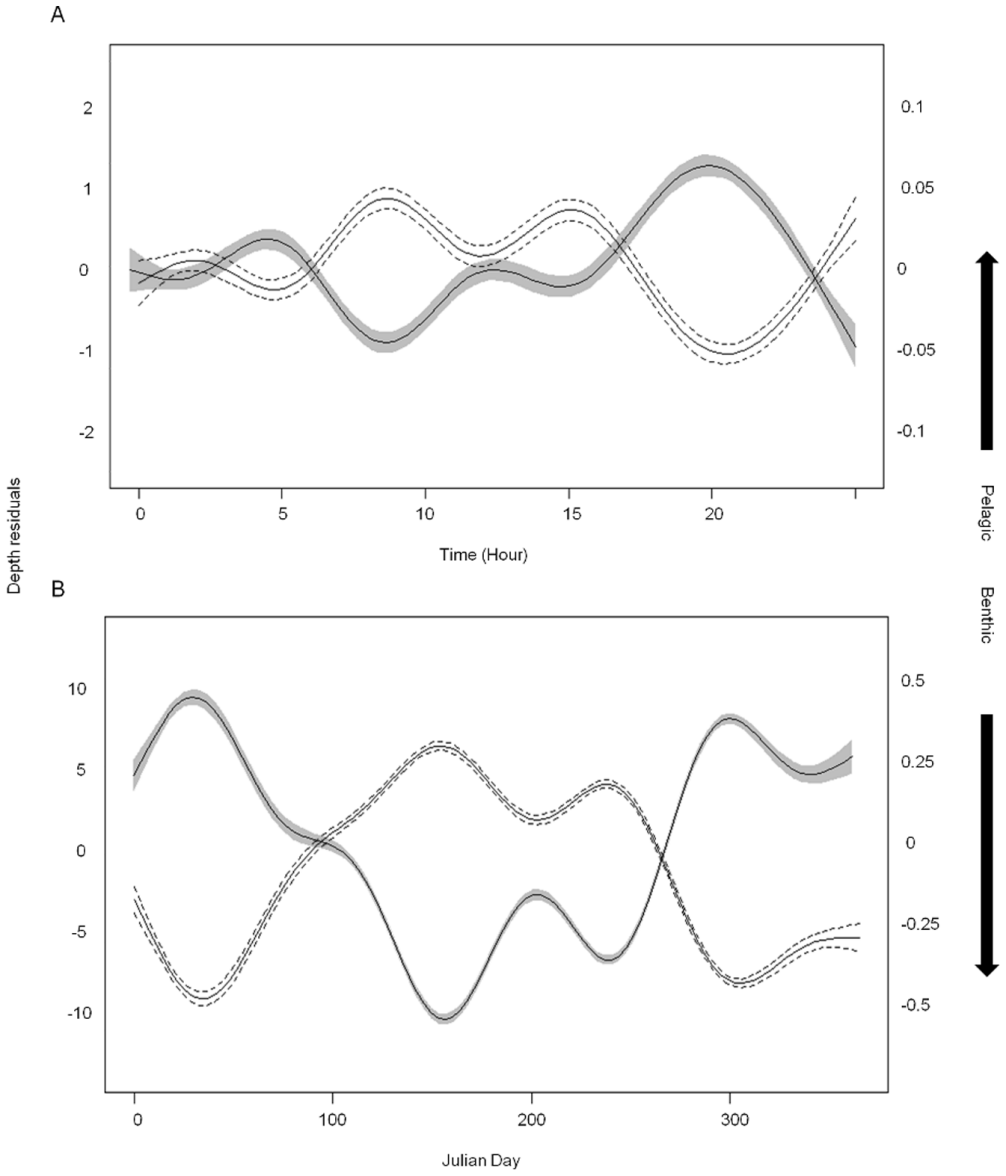
Passive telemetry: modelling of seasonal shifts in behaviour. Partial coefficients of Depth (black line - shaded 95% CI) and Behavioural State (black line - dashed 95% CI) as a function of (a) minutes across 24 hours and (b) daily values over 365 days from GAM models. The patterns show a strong positive correlation (r>0.9) such that when the fish are at deeper depths they are more likely to occupy a benthic state and vice versa.

The results of the spatio-temporal GAM models revealed changes in both depth and behavioural state of the fish although with different levels of variance explained (R^2^_adj_ = 0.84 and 0.15, respectively - Table S2). Fish were significantly deeper at St5 and shallowest at St2, and the likelihood of pelagic state was greatest at St4 and lowest at St5. Both models showed cyclical patterns across daily and annual scales that show a strong negative correlation with one another, i.e., fish were more likely to be in a benthic state when found deeper and *vice versa* (Figure 7). Daily patterns in the depth/state of the fish switched from deeper depths and benthic state during the night, with peaks in this behaviour near dawn (5am) and dusk (8pm), to shallower depths and pelagic behaviour during the day. However, the diel behaviour was not as strong as the shift in depth and behavioural state across a full year in both models. Interestingly, there seemed to be a seasonal switch from shallower benthic behaviour in winter months towards deeper and increasingly pelagic behaviour throughout summer. Animals then steadily shifted back as winter approached.

## Discussion

### Cycles and environmental limitations to vertical migration

Our results clearly show that vertical migratory behaviour is very common and significant in seamount-associated adult blackspot seabream. All four actively tracked and three passively monitored fishes vertically migrated at least on a daily basis during their monitoring periods, with individual depth displacements (vertical envelope) of up to 134 to 386 m representing an average 41% to 59% of maximum attained individual depths. Such migrations of several hundreds of meters compare in magnitude to those of better known pelagic marine predators, such as sharks [16], [17] and tunas [18], [19]. This is the first direct, documented evidence that resident seamount fish species undertakes substantial vertical migrations.

Blackspot seabream exhibited significant temporal patterns in their vertical migratory behaviour. Most noticeably, a diel pattern is clearly visible in the long-term passive telemetry time series. This is reflected by both the 24 hour peaks in the FFT analysis and the highly significant diel changes in the GAM modelling between deeper, day-time behaviour and shallower, dusk and early night-time behaviour. The same general diel vertical pattern was shown by the actively tracked fish, although this varied considerably between individuals. Positive vertical migration (deeper during the day, shallower during the night) has been previously reported for other pelagic predators and assumed as an evolutionary driving force for developing specific morphological and physiological adaptations that are believed to provide increased vision abilities under low light levels, such as a large visual retina or specific eye circulatory systems [20]. Blackspot seabream do have large eyes and associated retina that would provide increased vision in deepwater conditions during the day, when residual light can penetrate the ocean down to several hundreds of meters in oligotrophic environments such as the Azores. They are capable of detecting their prey in both benthic and pelagic environments [9] while also avoiding their benthopelagic predators, such as the conger eel [21]. Successfully and preferentially enacting these behaviours in low-light conditions could also explain why seabream go deeper at night during the full moon periods of increased illumination.

There were also important seasonal patterns in depth displacement, not only in the lower and upper ceilings attained by the passively tracked individuals but also in their dominant diel pattern. The average and maximum depths tended to increase towards summer and reduce towards winter, although not accompanied by a concomitant increase in minimum depth. Therefore, blackspot seabream experience a seasonal expansion in the vertical niche occupied at the Condor seamount. Again, such expansion may at least partially reflect the increased light penetration at deeper habitats during late summer and autumn, when the turbidity and productivity is lower [22].

Other than the indirect effect and limitations posed by light penetration at depth (see above), temperature, salinity and dissolved oxygen are major factors that can have a strong influence on the physiology and activity of fishes, and thus may affect the survival and shape the vertical habitat envelope of blackspot seabream. A major prediction of this hypothesis is that seabream, which are ectothermic, should remain below a certain temperature given that they are known to live and explore intermediate depths and temperatures in their adult phase [7]. Although difficult to predict, it is reasonable to assume that this threshold should be, at least, below the point where the rate of change of water temperature (hence internal temperature) is higher because that would pose additional physiological stress. In fact, both actively tracked and passively monitored fish were always detected under 130 m depth, well below the summer lower thermocline, and within a thermic envelope between 13.5 and 15.5°C except for occasionally slightly colder waters. More striking was the convergence between the minimum depth and the upper limit of the pycnocline at about 130 m; this was also where levels of dissolved oxygen dropped. It is possible that rather than the thermocline, it is actually the pycnocline that imposes a limit on the physiology of blackspot seabream and other deep-sea fishes.

### Vertical migrations and pelagic prey

We explored the possibility that the vertical migrations of blackspot seabream are timed to match the vertical availability of prey within a given habitat envelope. We tested this hypothesis by actively tracking fish and their major diet constituent (DSL components) in the water column above the Condor seamount during the summer. Results show that during the night seabream tend to spend time in the water column on top of the seamount at places where putative DSL patches densely aggregate. During the daytime, however, when the DSL descends, we could not detect the concomitant movement of the fish, even if they do tend to go deeper. Instead, this vertical migration seems to be accompanied by a switch in their behavioural mode, that is, fish become more benthic. The fact that blackspot seabream feeds both on benthic animals as well as the pelagic DSL community [9] supports the hypothesis that they feed mostly on benthic items during the day and on the pelagic DSL during the night. In fact, ROV filming has shown schools of seabream on top of the seamount very close to the substrate during the day [23], but the absence of studies analysing diel changes in their diet precludes a conclusive statement. Because the DSL is more densely aggregated at night it could well be that it only pays off to spend time swimming in the water column during this period, whereas during the day, when visual acuity is higher and the DSL less dense at tractable depths, it is more profitable to roam over the bottom and find benthic prey such as small fishes, sea urchins and sea stars that hide amongst the rocky reefs and deepwater corals of the seamount.

The Condor seamount is classified as a shallow or intermediate mound with the upper slopes penetrating into the euphotic layer. The mechanisms that drive the increased productivity of zooplankton above the summit of these seamounts is thought to be the advection of the vertically migrating zooplankton above the seamount during the night, effectively trapping them on the summit and preventing their descent into deeper waters [5], [24]. Yet, this hypothesis is complicated by the seasonal pattern indicated by the passively monitored fish. These fish showed a change in depth trend between day vs. night depths, i.e., that in the winter and early spring they are actually shallower during the day. While we would expect that there would have been a seasonal change in behaviour of the fish during these months, when primary [22] and secondary productivity [24] has dramatically decreased, the reason for an alternation of occupied depth ranges between night and day are not clear. Recent research exploring bioenergetics at seamount locations have suggested that the vertical flux of prey may be the dominant mechanism involved in sustaining the large biomass of seamount fishes as opposed to the advection of primary producers into the area [25]. Therefore, these findings suggest that the species may undergo a shift in its feeding-rest cycle [26] to increased daytime foraging to compensate for a decrease in available DSL food resources.

In conclusion, this study is the first to successfully document the fine scale vertical and horizontal movements of deep-sea fishes residing at seamounts. We found that adult blackspot seabream migrate vertically when at seamounts and that these patterns are cyclic both in the short-term (tidal, diel) as well as long-term (seasonal) scales. Furthermore, the concurrent fine-scale three-dimensional measurement of fish movements and backscatter indicates that this behaviour partially matches the availability of pelagic, vertically migrating prey. The emerging pattern points to more complex spatial behaviours than previously anticipated. Our results suggest that deep-sea fishes can not only alternate between areas within a seamount but also switch their diel behavioural mode, most likely in response to prey availability. Such adaptive behaviour would help explain how meso-predatory deep-sea fishes have successfully evolved to become key constituents of seamount and slope habitats, where the high seasonality of predator-prey interactions must play a pivotal role in survivorship.

## Supporting Information

Figure S1 **Active telemetry: raw positions.** The raw horizontal positions of the four blackspot seabream actively tracked at the Condor seamount. Positions are separated by day period (daytime-white circles, nighttime-black circles) and by tracking period (new moon-empty circles, full moon-filled circles).
(TIF)

Figure S2 **Active telemetry: home ranges.** The 3Dimensional KUDs for the 4 fish detected during the active tracking phase of the experiment. The colour coding for the 50% and 90% Kernel for each fish are also shown (see legend inset).
(TIF)

Figure S3 **Active telemetry: vertical behaviour versus potential prey.** The transverse profiles across transects 1 and 2 (see Figure 1) of the Condor seamount (shaded grey) for day and night during the full moon phase of the active tracking experiment. The NASC is binned into 10×100 m cells with higher backscatter values represented by darker red. Also shown is the vertical profile of the NASC with the maximum value found across transects at each 10 m vertical bin displayed. Symbols represent the individual detections made for each of the four individuals that were tracked (see legend).
(TIF)

Figure S4 **Active telemetry: vertical behaviour versus potential prey.** The Transverse profiles across transects 1 to 4 (see Figure 1) of the Condor seamount (shaded grey) for day and night during the new moon phase of the active tracking experiment. The NASC is binned into 10×100 m cells with higher backscatter values represented by darker red. Also shown is the vertical profile of the NASC with the maximum value found across transects at each 10 m vertical bin displayed. Symbols represent the individual detections made for each of the four individuals that were tracked (see legend).
(TIF)

Figure S5 **Passive telemetry: general trends.** Top panels: vertical depth detections for three of the monitored individuals. Detections are aggregated into columns representing detections made over a 24 hour period. Also shown is a 7 day moving average (red). Note: detections are aggregated across all stations including those at the temporary flanking stations for each individual. Lower panels: an abacus plot showing the raw detections at each of the seven stations (5 core and 2 flanking) across the detection period for the three individuals.
(TIF)

Figure S6 **Passive telemetry: differences in shallow versus deeper stations.** The average recorded depths and 95% CI errors for fish detections at the core stations (S1–S5) and the flanking, deeper stations (S6–S7) for the months between April and September 2011.
(TIF)

Figure S7 **Passive telemetry: fine scale temporal patterns.** Top panel: a time series of hourly depths for each of the three monitored individuals averaged across all months between April 2011 and Jan 2013. Middle panels: Fast-Fourier Transform generated periodogram for hourly depths. Peaks of a higher magnitude indicate periods that are the most dominant within the time series. The periodicities of the most important peaks are identified for each individual that show prominent peaks at 6 and 24 hours. Lower panel: the autocorrelation function (Acf) for each time series showing the lag correlation over each 24 hour period. Points above the dashed line indicate lags that are significantly correlated with each value.
(TIF)

Table S1 **Estimates of the three-dimensional Kernel Utilization Distributions (KUDs) for all four seabream actively tracked at the Condor seamount.** The percentage overlap between the individual KUDs is also shown on an overlap matrix: lower triangle for 50% centre of activity KUDs, upper triangle for 95% home range KUDs).
(DOCX)

Table S2 GAM results. Parameters of the two GAM that modelled the annual (day365) and short term (time) dynamics of vertical depth and behavioural state of the three monitored individuals across the five core stations (St1–St5). The coefficients of the parametric and smooth terms are shown. Significant terms are noted in bold.
(DOCX)

## References

[1] Rogers AD (1994) *The biology of seamounts* in *Advances Marine Biology*, 1994 (ed. JHS Blaxter and AS Southward) pp.305–350. London: academic press.

[2] Morato T, Clark MR (2007) Seamount fishes: ecology and life histories. In: Pitcher TJ, Morato T, Hart PJB, Clark MR, Haggan N, Santos RS (eds) *Seamounts: Ecology, Fisheries & Conservation*, Book 12. Blackwell: Oxford.

[3] Rowden AA, Dower JF, Schlacher TA, Consalvey M, Clark MR (2010) Paradigms in seamount ecology: fact, fiction and future. Mar. Ecol. 31: 226–241 10.1146/annurev-marine-120308-081109).

[4] Menezes GM, Giacomello E (2013) Spatial and temporal variability of demersal fishes at Condor Seamount (Northeast Atlantic). Deep-Sea Res II 98A: 101–113 10.1016/j.dsr2.2013.08.010).

[5] Colaço A, Giacomello E, Porteiro F, Menezes GM (2013) Trophodynamic studies on the Condor seamount (Azores, Portugal, North Atlantic). Deep-Sea Res. II 98A: 178–189 (doi.org/10.1016/j.dsr2.2013.01.010i)

[6] Afonso P, Graça G, Berke G, Fontes J (2012) First observations on seamount habitat use of blackspot seabream (Pagellus bogaraveo) using acoustic telemetry. J. Exp. Mar. Biol. Ecol. 1: 436–437 10.1016/j.jembe.2012.08.003).

[7] Menezes GM, Sigler MF, Silva HM, Pinho MR (2006) Structure and zonation of demersal fish assemblages off the Azores archipelago (mid-Atlantic). Mar. Ecol. Progr. Ser 324: 241–260 10.3354/meps324241).

[8] Sánchez F (1983) Biology and fishery of the red sea-bream (Pagellus bogaraveo, B.) in VI, VII and VIII subareas of ICES. ICES Council Meeting 1983.

[9] Morato T, Sola E, Grós MP, Menezes G (2001) Feeding habits of two congener species of seabreams, Pagellus bogaraveo and Pagellus acarne, off the Azores (northeastern Atlantic) during spring of 1996 and 1997. Bull. Mar. Sci. 69(3): 1073–1087 10.1093/hmg/ddn024).

[10] Morato T, Pitcher TJ, Clark M R, Menezes G, Tempera F, et al (2010) Can we protect seamount for research? A call for conservation. Oceanography 23(1): 190–199 10.5670/oceanog.2010.71).

[11] Tempera F, Giacomello E, Mitchell N, Campos AS, Braga Henriques A, et al. (2012) Mapping the Condor seamount seafloor environment and associated biological assemblages (Azores, NE Atlantic). In: Harris PT, Bakker EK (eds) *Seafloor Geomorphology as Benthic Habitat*. Geohab Atlas.

[12] Afonso P, Fontes J, Holland KN, Santos RS (2009) Multi-scale patterns of habitat use in a highly mobile reef fish, the white trevally Pseudocaranx dentex, and their implications for marine reserve design. Mar. Ecol. Prog. Ser. 381: 273–286 10.3354/meps07946)

[13] Jarvis ET, Lowe CG (2008) The effects of barotrauma on the catch-and-release survival of southern California nearshore and shelf rockfish (Scorpaenidae, Sebastes spp.). Can. J. Fish. Aquat. Sci. 65: 1286–1296 10.1139/F08-071)

[14] Maclennan DN, Fernandes PG, Dalen J (2002) A consistent approach to definitions and symbols in fisheries acoustics. ICES J. Mar. Sci. 59(2): 365–369 10.1006/jmsc.2001.1158.sthash.nAtdasWs.dpuf))

[15] Simpfendorfer CA, Olsen EM, Heupel MR, Moland E (2012) Three-dimensional kernel utilization distributions improve estimates of space use in aquatic animals. Can. J. Fish. Aquat Sci 69(3): 565–572 10.1139/f2011-179)

[16] Nakano H, Matsunaga H, Okamoto H, Okazaki M (2003) Acoustic tracking of bigeye thresher shark Alopias superciliosus in the eastern Pacific Ocean. Mar. Ecol. Prog. Ser. 265: 255–261 10.3354/meps265255)

[17] Stevens JD, Bradford RW, West GJ (2010) Satellite tagging of blue sharks (Prionace glauca) and other pelagic sharks off eastern Australia: depth behaviour, temperature experience and movements. Mar. biol. 157(3): 575–591 10.1007/s00227-009-1343-6)

[18] Holland KN, Brill RW, Chang RK, Sibert, JR, Fournier DA (1992) Physiological and behavioural thermoregulation in bigeye tuna (Thunnus obesus). Nature 358: 410–412 10.1038/358410a0) 1641023

[19] Dagorn L, Bach P, Josse E (2000) Movement patterns of large bigeye tuna (Thunnus obesus) in the open ocean, determined using ultrasonic telemetry. Mar. Biol. 136(2): 361–371 10.1007/s002270050694)

[20] Weng KC, Block BA (2004) Diel vertical migration of the bigeye thresher shark (Alopias superciliosus), a species possessing orbital retia mirabilia. Fish. Bull. 102(1): 221–229.

[21] Morato T, Solà E, Grós MP, Menezes GM (1999) Diets of forkbeard (Phycis phycis) and conger eel (Conger conger) off the Azores during spring of 1996 and 1997. Arquipélago. Life Mar. Sci. 17A: 51–64 10.1111/j.1439-0426.2010.01467.x.)

[22] Santos M, Moita MT, Bashmachnikov I, Menezes GM, Carmo V, et al (2013) Phytoplankton Variability and Oceano- graphic Conditions at Condor Seamount, Azores (NE Atlantic). Deep Sea Res. II. 98A: 52–62 (doi.org/10.1016/j.dsr2.2013.05.037)

[23] Porteiro FM, Gomes-Pereira JN, Pham CK, Tempera F, Santos RS (2013) Distribution and habitat association of benthic fish on the Condor seamount (NE Atlantic, Azores) from in situ observations. Deep-Sea Res. II. 98A: 114–128 10.1016/j.dsr2.2013.09.015i)

[24] Carmo V, Santos M, Menezes GM, Loureiro C, Lambardi P, Martins A (2013) Variability of zooplankton communities at Condor seamount and surrounding areas, Azores (NE Atlantic). Deep-Sea Res. II. 98A: 63–74 10.1016/j.dsr2.2013.08.007i)

[25] Morato T, Bulman C, Pitcher TJ (2009) Modelled effects of primary and secondary production enhancement by seamounts on local fish stocks. Deep-Sea Res. II. 56(25): 2713–2719.

[26] Genin A (2004) Bio-physical coupling in the formation of zooplankton and fish aggregations over abrupt topographies. J. Mar. Sys. 50: 3–20 10.1111/j.1096-0031.2009.00286.x..).

