# Supplementary figures and images for: Vertical Migrations of a Deep-Sea Fish and Its Prey
Source: PLoS One. 2014 May 23;9(5):e97884. doi: 10.1371/journal.pone.0097884 (PMC4032296; doi:10.1371/journal.pone.0097884)

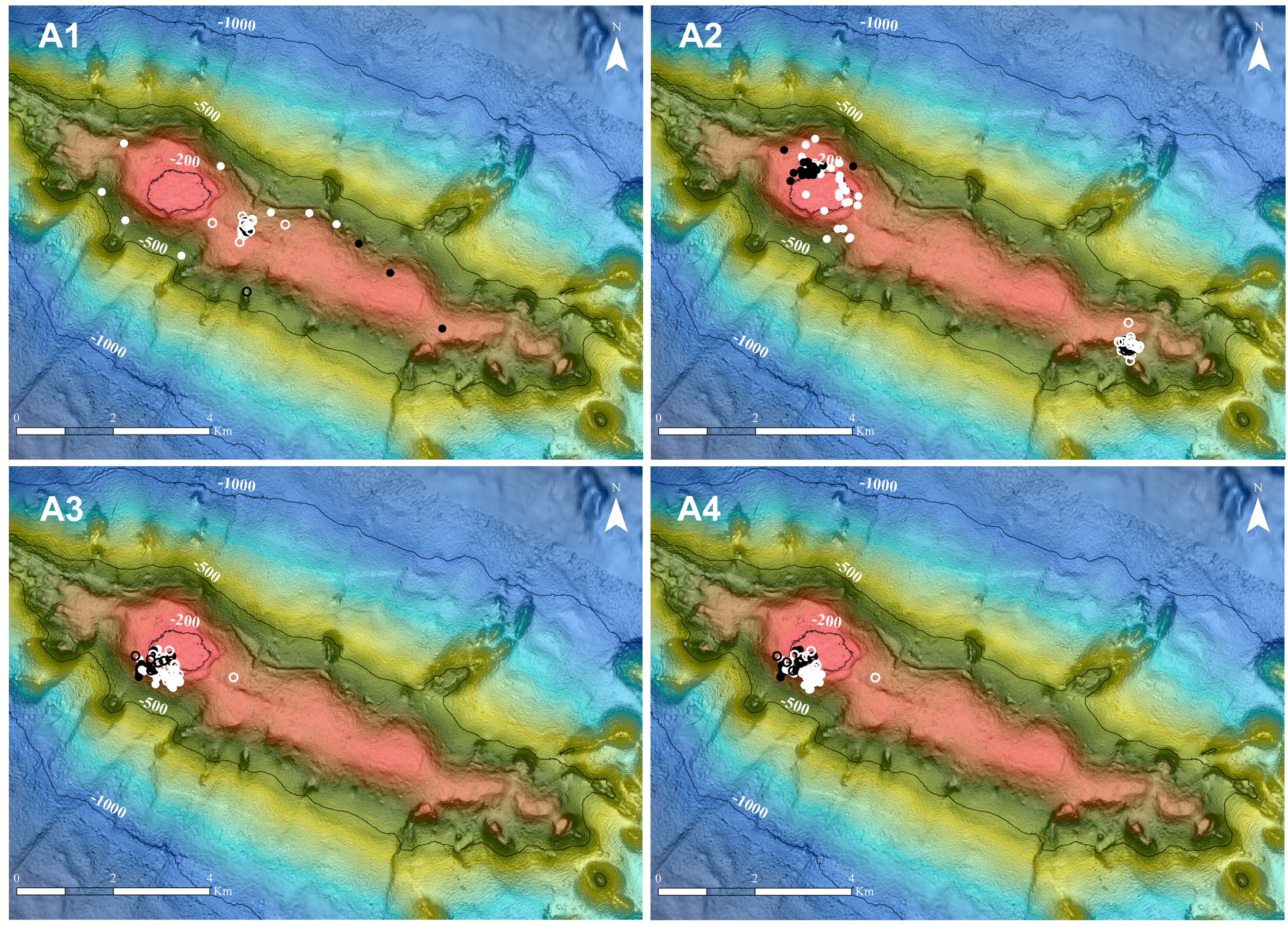

Supplement: Figure S1 — Active telemetry: raw positions. The raw horizontal positions of the four blackspot seabream actively tracked at the Condor seamount. Positions are separated by day period (daytime-white circles, nighttime-black circles) and by tracking period (new moon-empty circles, full moon-filled circles). (TIF) [file pone.0097884.s001.tif]

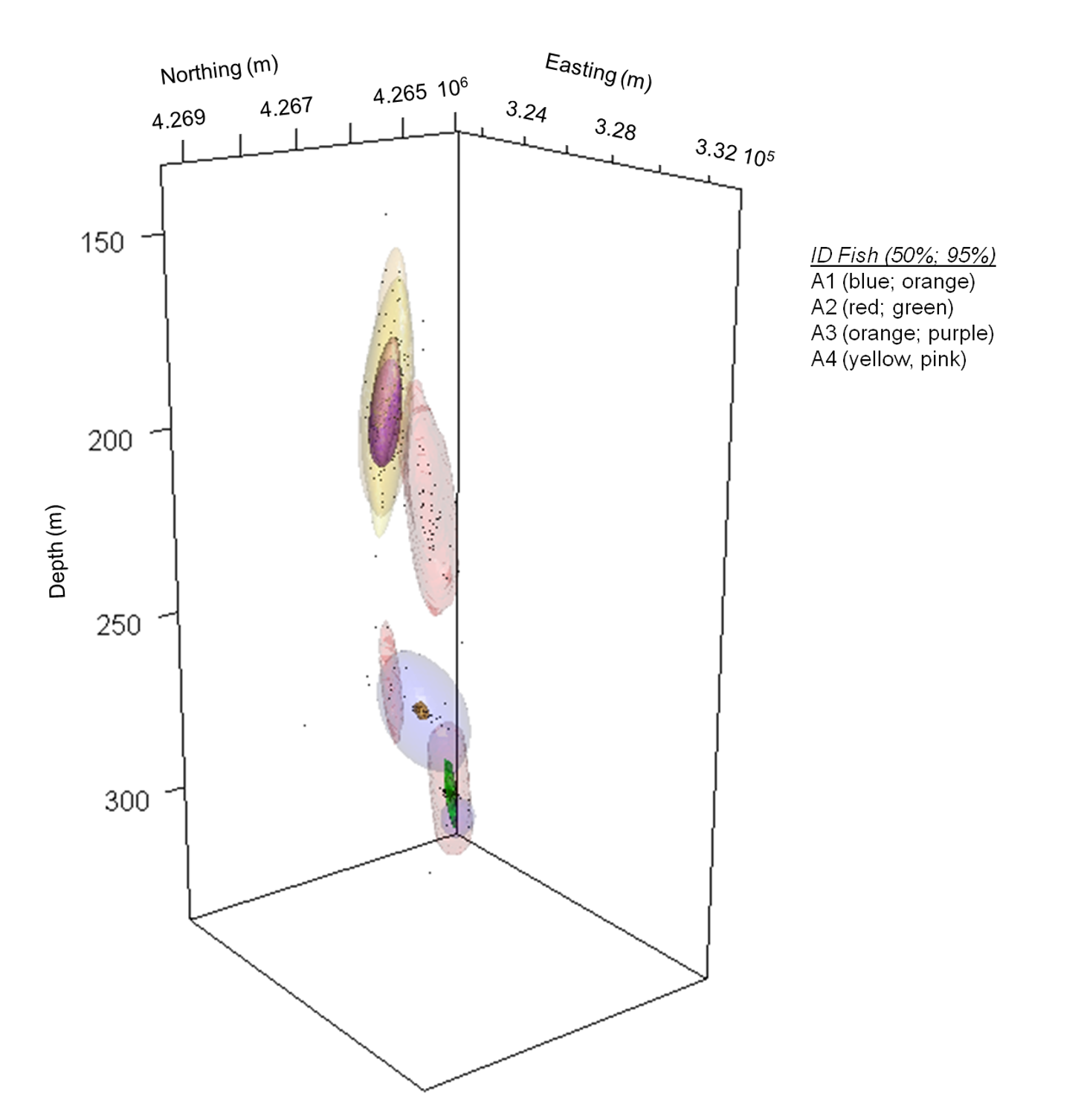

Supplement: Figure S2 — Active telemetry: home ranges. The 3Dimensional KUDs for the 4 fish detected during the active tracking phase of the experiment. The colour coding for the 50% and 90% Kernel for each fish are also shown (see legend inset). (TIF) [file pone.0097884.s002.tif]

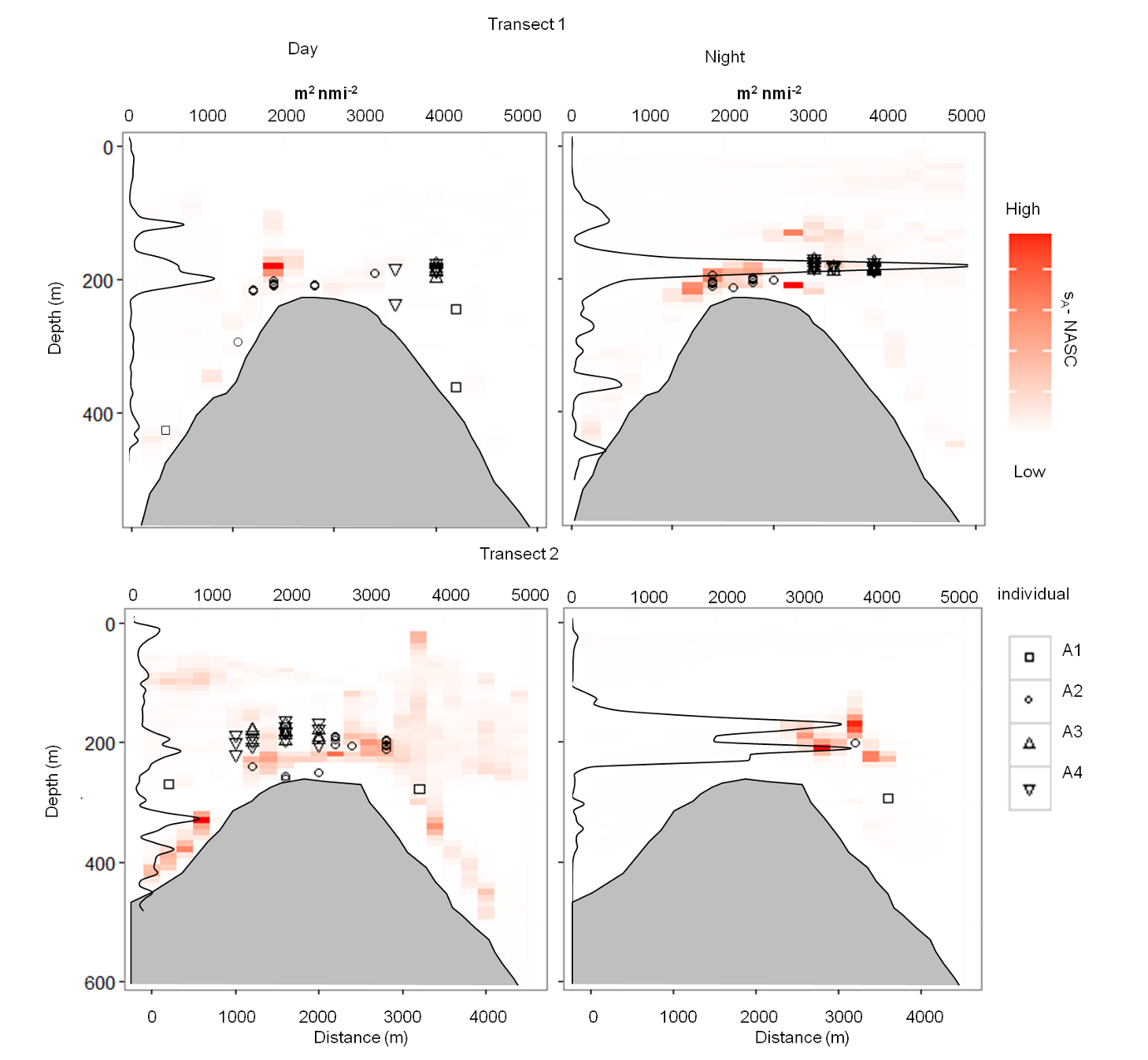

Supplement: Figure S3 — Active telemetry: vertical behaviour versus potential prey. The transverse profiles across transects 1 and 2 (see Figure 1) of the Condor seamount (shaded grey) for day and night during the full moon phase of the active tracking experiment. The NASC is binned into 10×100 m cells with higher backscatter values represented by darker red. Also shown is the vertical profile of the NASC with the maximum value found across transects at each 10 m vertical bin displayed. Symbols represent the individual detections made for each of the four individuals that were tracked (see legend). (TIF) [file pone.0097884.s003.tif]

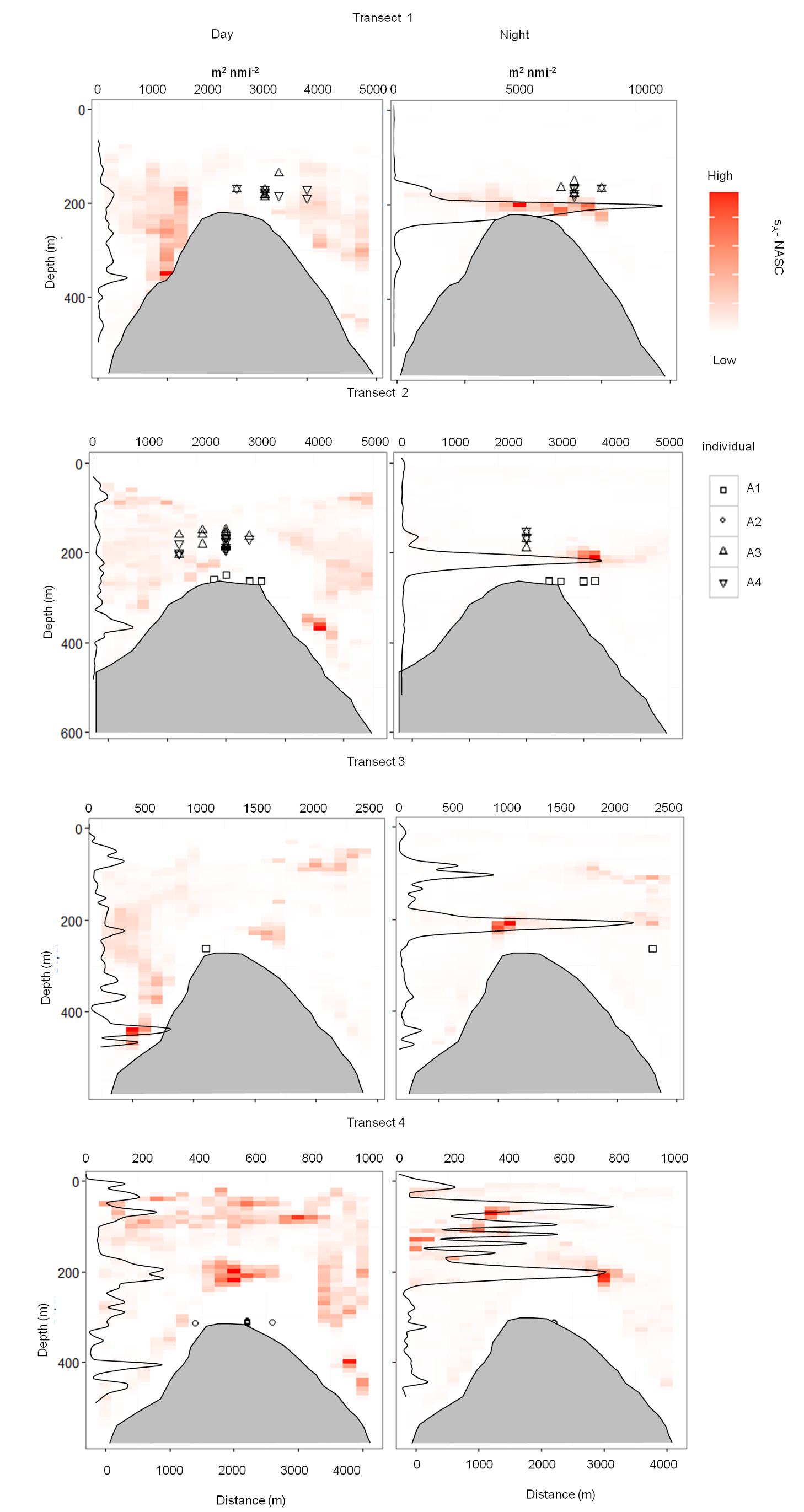

Supplement: Figure S4 — Active telemetry: vertical behaviour versus potential prey. The Transverse profiles across transects 1 to 4 (see Figure 1) of the Condor seamount (shaded grey) for day and night during the new moon phase of the active tracking experiment. The NASC is binned into 10×100 m cells with higher backscatter values represented by darker red. Also shown is the vertical profile of the NASC with the maximum value found across transects at each 10 m vertical bin displayed. Symbols represent the individual detections made for each of the four individuals that were tracked (see legend). (TIF) [file pone.0097884.s004.tif]

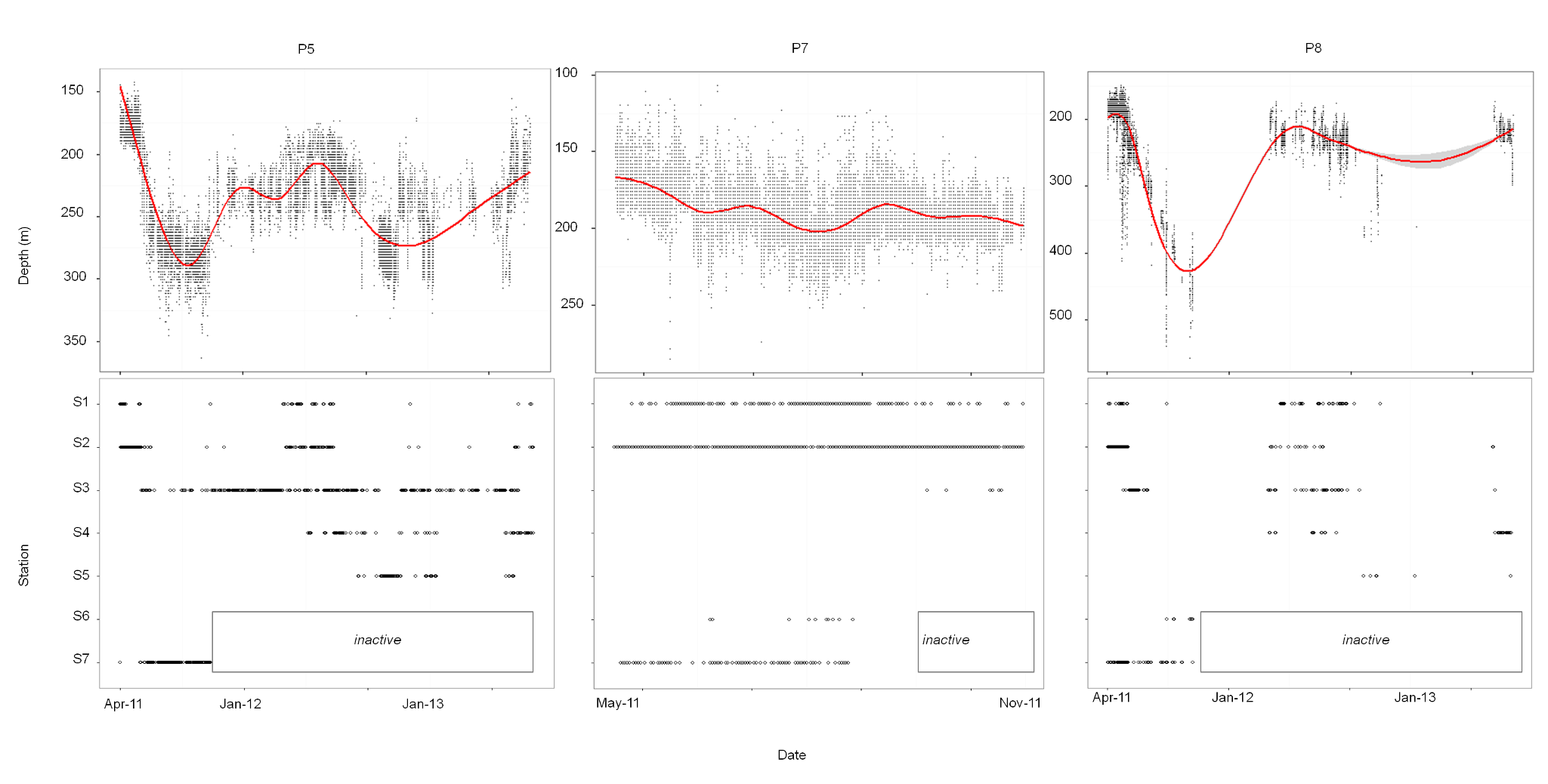

Supplement: Figure S5 — Passive telemetry: general trends. Top panels: vertical depth detections for three of the monitored individuals. Detections are aggregated into columns representing detections made over a 24 hour period. Also shown is a 7 day moving average (red). Note: detections are aggregated across all stations including those at the temporary flanking stations for each individual. Lower panels: an abacus plot showing the raw detections at each of the seven stations (5 core and 2 flanking) across the detection period for the three individuals. (TIF) [file pone.0097884.s005.tif]

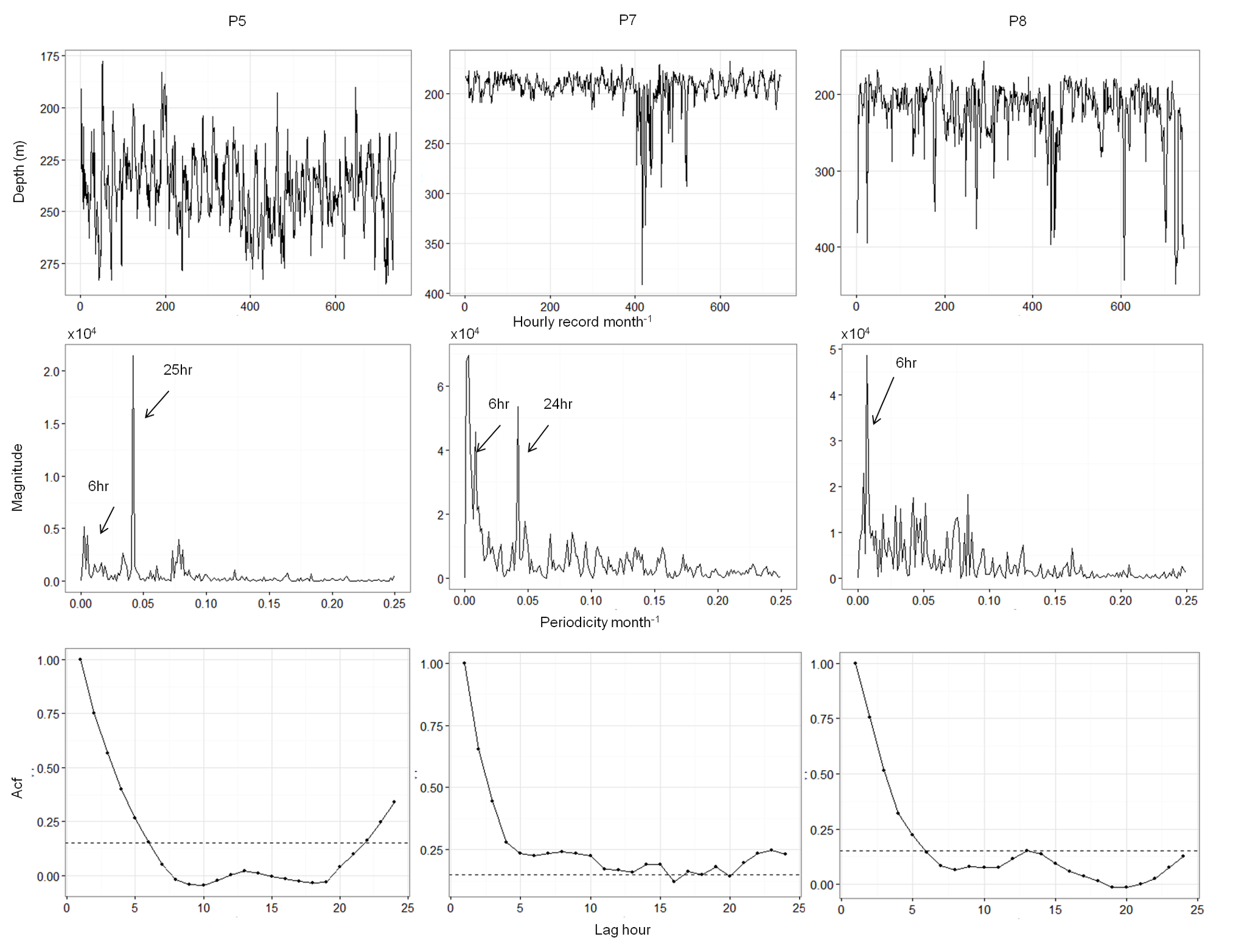

Supplement: Figure S7 — Passive telemetry: fine scale temporal patterns. Top panel: a time series of hourly depths for each of the three monitored individuals averaged across all months between April 2011 and Jan 2013. Middle panels: Fast-Fourier Transform generated periodogram for hourly depths. Peaks of a higher magnitude indicate periods that are the most dominant within the time series. The periodicities of the most important peaks are identified for each individual that show prominent peaks at 6 and 24 hours. Lower panel: the autocorrelation function (Acf) for each time series showing the lag correlation over each 24 hour period. Points above the dashed line indicate lags that are significantly correlated with each value. (TIF) [file pone.0097884.s007.tif]
