# Supplementary material for: Vertical Migrations of a Deep-Sea Fish and Its Prey
Source: PLoS One. 2014 May 23;9(5):e97884. doi: 10.1371/journal.pone.0097884 (PMC4032296; doi:10.1371/journal.pone.0097884)
Supplement: Table S1 — Estimates of the three-dimensional Kernel Utilization Distributions (KUDs) for all four seabream actively tracked at the Condor seamount. The percentage overlap between the individual KUDs is also shown on an overlap matrix: lower triangle for 50% centre of activity KUDs, upper triangle for 95% home range KUDs). (DOCX) [file pone.0097884.s008.docx]

**Supporting information table S1**

**Table S1 – estimates of the three-dimensional Kernel Utilization Distributions (KUDs) for all four seabream actively tracked at the Condor seamount.** The percentage overlap between the individual KUDs is also shown on an overlap matrix: lower triangle for 50% centre of activity KUDs, upper triangle for 95% home range KUDs).

| Fish ID | 50% KUD (m^3^) | 95% KUD (m^3^) | % overlap | | | |  |
| --- | --- | --- | --- | --- | --- | --- | --- |
|  |  |  |  | | | |  |
|  |  |  | A1 | A2 | A3 | A4 | |
| A1 | 8.4 x10^-5^ | 8.9 x10^-7^ | - | 2.2 | 26.1 | 38 | |
| A2 | 6.7 x10^-6^ | 1.7 x10^-8^ | 0 | - | 16 | 20 | |
| A3 | 6.8 x10^-6^ | 4.3 x10^-7^ | 0 | 0 | - | 60 | |
| A4 | 4.1 x10^-4^ | 1.1 x10^-6^ | 0 | 0 | 2 | - | |
