# Supplementary material for: Vertical Migrations of a Deep-Sea Fish and Its Prey
Source: PLoS One. 2014 May 23;9(5):e97884. doi: 10.1371/journal.pone.0097884 (PMC4032296; doi:10.1371/journal.pone.0097884)
Supplement: Table S2 — GAM results. Parameters of the two GAM that modelled the annual (day365) and short term (time) dynamics of vertical depth and behavioural state of the three monitored individuals across the five core stations (St1–St5). The coefficients of the parametric and smooth terms are shown. Significant terms are noted in bold. (DOCX) [file pone.0097884.s009.docx]

**Supporting information table S2**

**Table S2 –** **GAM results**. Parameters of the two GAM that modelled the annual (day365) and short term (time) dynamics of vertical depth and behavioural state of the three monitored individuals across the five core stations (St1-St5). The coefficients of the parametric and smooth terms are shown. Significant terms are noted in bold.

| **parametric** | model | | | |
| --- | --- | --- | --- | --- |
|  | state  coeff^se^ t | | depth  coeff^se^ t | |
| intercept (CE) | 0.34^0.02^ | **-14.37** | 229.7^0.14^ | **1704** |
| NW | -0.06^0.04^ | **-16.65** | -16.1^0.19^ | **-83.8** |
| W | 0.38^0.03^ | -1.77 | -45.2^0.15^ | **-282.6** |
| E | 0.72^0.04^ | **-3.71** | -21.9^0.25^ | **-89.2** |
| SE | -0.14^0.02^ | **13.43** | 53.5^0.21^ | **246.6** |
| **Smooth terms** | est. df | Chi sq. | est. df | Chi sq. |
|  | | | | |
| time (dec. min)  day365 | 8.9  9.0 | **562.4**  **7217** | 8.9  9.0 | **69.8**  **939.7** |
| n = 66919  R^2^ = 0.15 0.84 | | | | |
